# Classification of Breast Cancer Cells Using the Integration of High-Frequency Single-Beam Acoustic Tweezers and Convolutional Neural Networks

**DOI:** 10.3390/cancers12051212

**Published:** 2020-05-12

**Authors:** Hae Gyun Lim, O-Joun Lee, K. Kirk Shung, Jin-Taek Kim, Hyung Ham Kim

**Affiliations:** 1 Future IT Innovation Laboratory, Pohang University of Science and Technology, Pohang 37673, Korea; haegyun@postech.ac.kr (H.G.L.); ojlee112358@postech.ac.kr (O.-J.L.); 2 Department of Biomedical Engineering, University of Southern California, Los Angeles, CA 90089, USA; kkshung@usc.edu; 3 Department of Creative IT Engineering, Pohang University of Science and Technology, Pohang 37673, Korea

**Keywords:** cancer cell classification, convolutional neural networks, single-beam acoustic tweezers, high-frequency ultrasound, cell deformation

## Abstract

Single-beam acoustic tweezers (SBAT) is a widely used trapping technique to manipulate microscopic particles or cells. Recently, the characterization of a single cancer cell using high-frequency (>30 MHz) SBAT has been reported to determine its invasiveness and metastatic potential. Investigation of cell elasticity and invasiveness is based on the deformability of cells under SBAT’s radiation forces, and in general, more physically deformed cells exhibit higher levels of invasiveness and therefore higher metastatic potential. However, previous imaging analysis to determine substantial differences in cell deformation, where the SBAT is turned ON or OFF, relies on the subjective observation that may vary and requires follow-up evaluations from experts. In this study, we propose an automatic and reliable cancer cell classification method based on SBAT and a convolutional neural network (CNN), which provides objective and accurate quantitative measurement results. We used a custom-designed 50 MHz SBAT transducer to obtain a series of images of deformed human breast cancer cells. CNN-based classification methods with data augmentation applied to collected images determined and validated the metastatic potential of cancer cells. As a result, with the selected optimizers, precision, and recall of the model were found to be greater than 0.95, which highly validates the classification performance of our integrated method. CNN-guided cancer cell deformation analysis using SBAT may be a promising alternative to current histological image analysis, and this pretrained model will significantly reduce the evaluation time for a larger population of cells.

## 1. Introduction

Image analysis of cancer cells is an emerging technique with growing applications in cancer research and plays a vital role in accurate diagnoses of cancer [1,2,3,4]. Histological image analysis has been extensively studied as a clinical diagnostic method of primary cancer cell classification after biopsy. However, due to the huge variability of the quality of/the condition of histological images and the subjective nature of manual analysis by experts, several limitations have been found [5,6,7]. One limitation, common to all manual image analysis, is observational variation among histopathologists and clinicians. Another critical limitation is non-automatic complex analysis protocols that increase evaluation times without increasing reliability. To overcome those major hurdles, an accurate and reliable quantitative analysis method that directly measures the physical properties of cells is gaining attention rapidly.

Over the years, there have been numerous studies that measured cell biomechanics using various techniques such as atomic force microscopy (AFM) [8,9,10], optical tweezers (OT) [11,12], magnetic tweezers [13], and stretchable substrates [14]. It is well documented that cell elasticity is closely linked to the invasion potential and infectibility of cells. However, the intrinsic limitations of those techniques, such as direct contact, limited forces, or labeling inside cells hinder reliable measurement at a single-cell level [15]. Among various attempts to measure cell elasticity, approaches using single-beam acoustic tweezers (SBAT) have emerged as a promising tool due to its micrometer-sized trapping, non-contact exertion of the force, no labeling requirement, and nanonewton trapping forces [16,17,18,19,20,21]. In the last decade, there has been tremendous success in studying the characteristics of a single cell using high-frequency ultrasound stimulation and acoustic tweezers [22]. In a study by SBAT, a 200 MHz ultrasonic transducer measured calcium responses of cultured breast cancer cells using the local cell membrane deformation [20], and the pattern of deformability of various cancer cells was analyzed for identifying cancer cell invasiveness [23].

In this study, experiments for cancer cell identification are presented using a high-frequency SBAT system that offers micrometer resolution spatially. This system is based on trapping and deforming a single cell using acoustic forces and quantifies the degree of the deformation caused by SBAT. Contrary to other tweezer techniques, SBAT can generate trapping forces up to a few hundred nanonewtons and can press the cell against the wall [17,24,25,26]. The level of cell deformation can be controlled by the input acoustic parameters. Usually, acoustic pressures lower than 1.0 MPa does not cause significant effects on the cell condition, as previously proven by live-cell viability tests [27,28,29]. An earlier examination of the cell deformation with the SBAT on and off was based on the extraction of boundaries of the cell image. The difference was clearly visible and was identified with boundary markings, but such manual analysis methods are time-consuming and are prone to subjective interpretation. The highly variable shape and structure of cells, as well as the variable locations of cell abnormalities, pose further challenges. The development of computational imaging analysis that minimizes variability and subjective analysis is of utmost importance.

A convolutional neural network (CNN) offers a better solution than previous manual analysis of cancer cell invasiveness. Using numerous convolutional filters, CNN can compare cell deformability in images with the SBAT on and off. Since CNN can train the optimal filters for classifying cancer cells, we can obtain much higher accuracy than the conventional handcrafted filters. The accuracy of CNN can be improved continuously as new cases are added.

The present study demonstrates the fabrication of a highly focused ultrasonic transducer at 50 MHz, the cell deformation phenomenon based on the radiation and trapping force of the SBAT, and the investigation of cell invasiveness using CNN. Two cell lines with different degrees of metastasis: MDA-MB-231 (highly invasive) and MCF-7 (weakly invasive) were deformed under the SBAT. For analysis of the deep learning CNN model, cell images were preprocessed to emphasize cell boundaries and reduce noise. CNN model was then trained to classify the images as MDA-MB-231 and MCF-7. The proposed model has shown significant accuracy (precision: 0.96, recall: 0.99, $F_{1}$ measure: 0.97). Derived values of cell membrane deformation under the static state demonstrate the capability of classification of human breast cancer cells. The integration of ultrasonic devices and CNN models may serve as meaningful groundwork offering a high precision rate for the development of a new diagnostic approach for cancel cell classification.

## 2. Results

**Figure 1:**
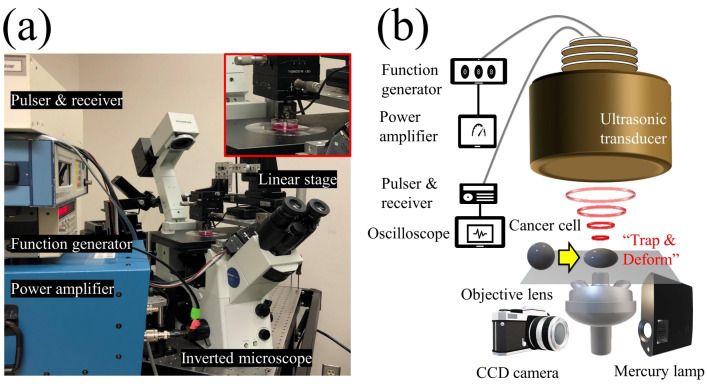
Schematic diagram of the experimental system. (**a**) Photograph of the experimental system. (**b**) The single-beam acoustic tweezers (SBAT) was driven at 50 MHz by sinusoidal bursts from a function generator amplified with a 50 dB amplifier. A single cell or a single sphere could be deformed by the SBAT.

**Figure 2:**
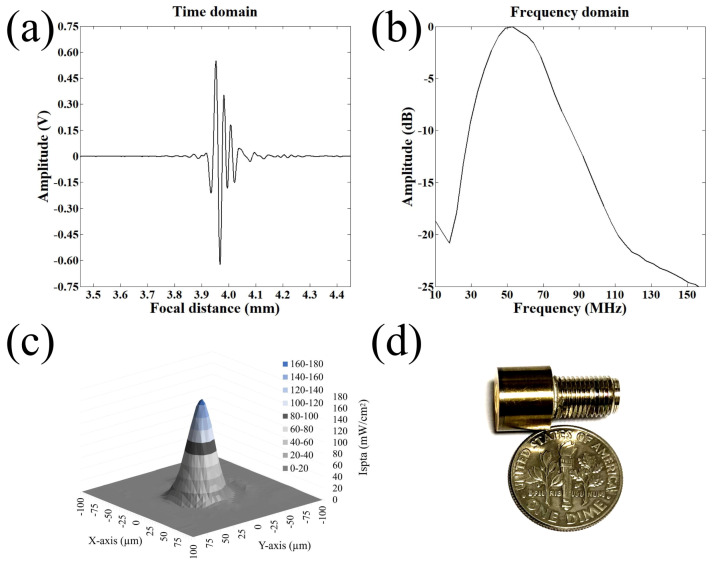
Fabrication of a highly focused 50 MHz transducer. (**a**) Receive-echo response. (**b**) Frequency spectrum. (**c**) 2D acoustic intensity field of spatial peak temporal average (ISPTA) was measured after a 50 MHz transducer was excited with the input parameters of $V_{pp}$ = 25 V, cycle numbers of 10, and pulse repetition frequency of 1 kHz. Acoustic pressure field of the ultrasonic transducers measured by a needle hydrophone. The −3 dB lateral beam width was measured to be 32 μm. (**d**) A photograph of the 50 MHz transducer.

Highly invasive and weakly invasive cancer cells have been implicated in different forms of metastatic potential, so numerous in-depth studies have investigated the invasiveness properties of cancer cells using various tools. The major challenges were related to cell safety issues caused by mechanical contact and to limited forces they can generate. On the contrary, SBAT with the benefit of having micro-trapping and strong-trapping force, can trap and press the cell leading to deformation along the transverse axis as depicted in Figure 1. For single-cell deformation, a focused ultrasonic transducer with a beam width comparable to a cell diameter was fabricated. Detailed profiles of the final product are demonstrated in Figure 2.

**Figure 3:**
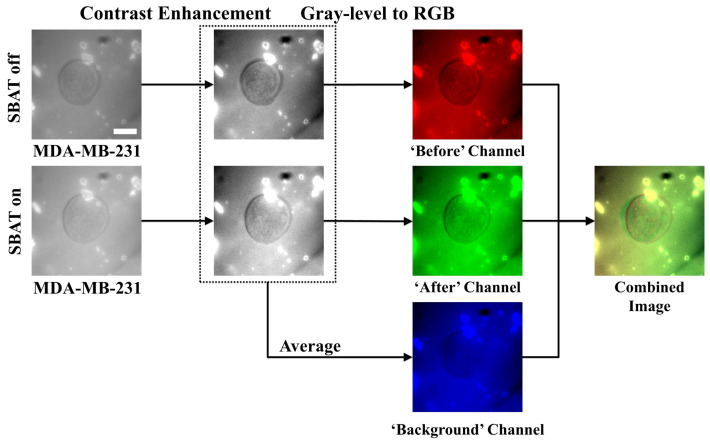
Preprocessing procedures for cell images. The first column presents photomicrographs taken when the SBAT was on and off, displaying the cell membrane deformation with the SBAT. The second column shows the results of the contrast enhancement. In the third column, we composed color channels of the combined image by using the enhanced photomicrographs. First, the red channel corresponds to the predeformation. The green channel exhibits the post deformation. As an average of the red and green channels, the blue channel will show us background areas. The last column is the result of the combination. We applied the same preprocessing method to both more and less deformed cells (MDA-MB-231 and MCF-7). Scale bar indicates 10 μm.

**Figure 4:**
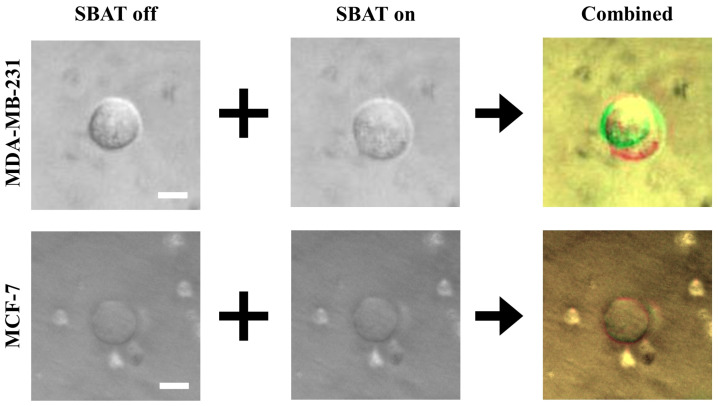
A comparison of the original photomicrographs with the preprocessed images. The first and second rows present preprocessing results of MDA-MB-231 and MCF-7, respectively. On the original images, the deformation is not always as significant as distinguishable with the naked eye. The preprocessing makes the deformation more distinctive than the original images. Additionally, the photomicrographs can include noises. On the combined images, only cell boundaries are emphasized by red and green colors, and noises have similar tones to the backgrounds. This point will provide robustness to the quality of photomicrographs. Scale bars indicate 10 μm.

**Figure 5:**
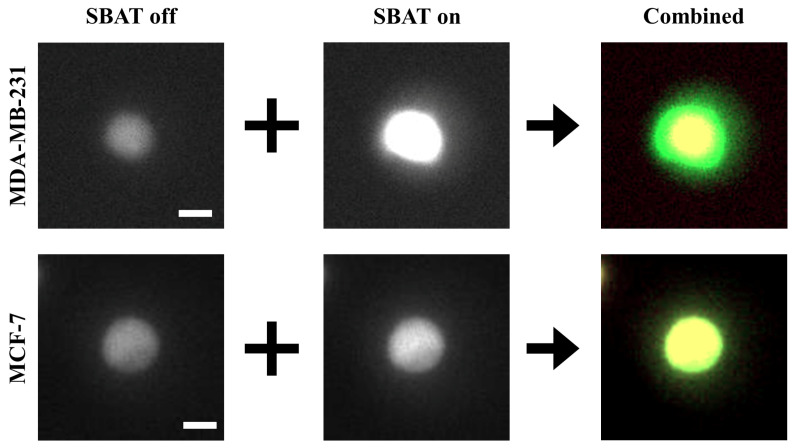
Preprocessing results for fluorescence cell images. When we use the fluorescence dyes, the differences between MDA-MB-231 and MCF-7 are more distinctive than the non-fluorescence ones. The fluorescence cell images barely include noises. These images mostly have black backgrounds. Therefore, as shown in the third column, yellow areas indicate cells before the deformation, and green areas mark changes caused by the deformation. Scale bars indicate 10 μm.

Biophysical characteristics i.e., the elasticity of cells and accurate detection of their morphological changes, can serve as a new diagnostic approach for cancer conditions. We investigated MDA-MB-231 (higher metastatic potential) and MCF-7 (weaker metastatic potential) in a suspended state floating above the Petri dish and monitored the cell lines during the SBAT. Increasing acoustic power facilitates cell deformation, causing area changes that are directly proportional to the applied acoustic pressure. Acoustic pressure was gradually increased from 0.0 to 1.0 MPa with the driving conditions of the fixed duty factor of 1%, the fixed pulse repetition frequency (PRF) of 1 kHz, and various peak-to-peak input voltages ($V_{pp}$). As shown in Figure 3, the MDA-MB-231 was imaged with the SBAT on and off. Figure 4 presents the comparison between the shapes of the MDA-MB-231 and MCF-7 with the SBAT on and off. Figure 5 is an example of fluorescence live-cell images. A similar tendency was shown for both cells; however, MDA-MB-231 still exhibited more deformation properties under the SBAT, which validates that the Young’s modulus of MDA-MB-231 cells was lower than that of the MCF-7 [9,30,31,32,33,34,35].

Previously, the deformability of a human breast cancer cell was measured relatively with an acoustic trap. The researchers still required manual analysis to track the change in the area of the cell after SBAT was turned on, drawing out the boundaries of the cell before and after deformation [23]. In this study, we developed a classification method using CNN to distinguish whether cells are highly or weakly invasive. For fast, precise, and automatic classification and detection of cancer cells after the SBAT experiment, we applied the CNN model to 40 cells that consist of 20 MDA-MB-231 and 20 MCF-7 cells.

**Figure 6:**
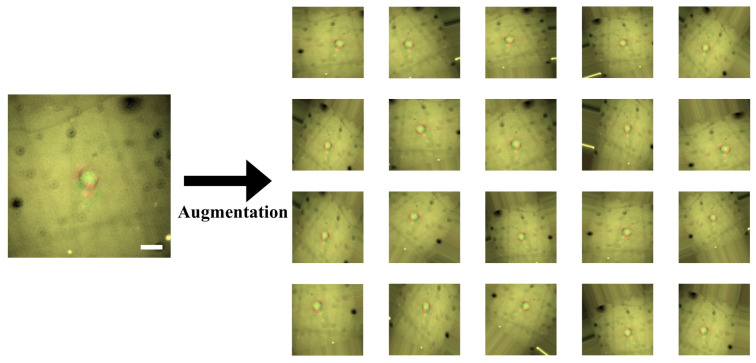
Data augmentation for combined cell images. We have only a relatively small-scaled dataset. Scales and resolutions of the images cannot be constant in reality. To make our convolutional neural network (CNN) model robust to this issue, we augmented the images by the parallel translation, rotational translation, scale adjustment, etc. Scale bars indicate 20 μm.

We conducted five-fold cross-validation. Each validation case used 80% of the cells for training the model and the remaining 20% for testing. All the cells were used as a testing sample once. By augmenting cell images, we generated 200 images for each cell (total 8000 images), as shown in Figure 6. Therefore, in a validation case, the CNN model was trained for classifying 6432 (32 × 201) cell images into invasive and non-invasive ones that include 3216 (16 × 201) images, respectively. Then, the model was tested by classifying the remaining 1608 (8 × 201) cell images according to their invasiveness. We measured the accuracy of the model on each validation case and evaluated the model using its average accuracy and variance for all the validation cases. The variance will show whether the proposed model can exhibit reliable performance generally.

**Figure 7:**
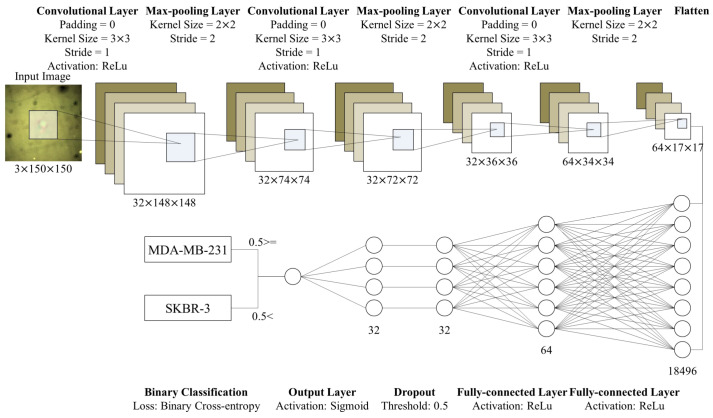
Structure of the proposed CNN model. This model consists of three convolutional layers and two FC layers. After each convolutional layer, we place max-pooling layers. We flatten outputs of the convolutional part and put it into the FC layers. After the first FC layer, we conduct dropout with a threshold, 0.5. Then, the output layer (the second FC layer) prints a single value in [0,1]. Based on the value, we discriminate whether cells in the input images are significantly deformed or not.

Our CNN model consists of three two-dimensional convolutional layers, three max-pooling layers, and two FC layers as shown in Figure 7. We implemented the model using Keras in Python. To evaluate the performance of our model, we used four metrics: accuracy (*a*), precision (*p*), recall (*r*), and $F_{1}$ measure ($F_{1}$). When *M* is a set of the automatically detected MDA-MB-231 cells, and $M^{*}$ denotes the actual MDA-MB-231 cells, the metrics can be formulated as:

(1) $$\begin{array}{c} a=\frac{\left|M^{*}\cap M\right|+\left|{\left(M^{*}\cup M\right)}^{c}\right|}{\left|U\right|},p=\frac{\left|M^{*}\cap M\right|}{\left|M\right|},r=\frac{\left|M^{*}\cap M\right|}{\left|M^{*}\right|},F_{1}=\frac{2pr}{p+r} \end{array}$$

where |·| denotes the size of sets, and *U* refers to all the cells in our dataset. The precision indicates a ratio of what we correctly found for what we found, the recall means a ratio of what we correctly found for what we should find, and $F_{1}$ measure is their harmonic mean. The CNN model contains various hyper-parameters. To determine the parameters, we conducted a grid search. Table 1 presents the ranges and step sizes of the search for each parameter.

**Figure 8:**
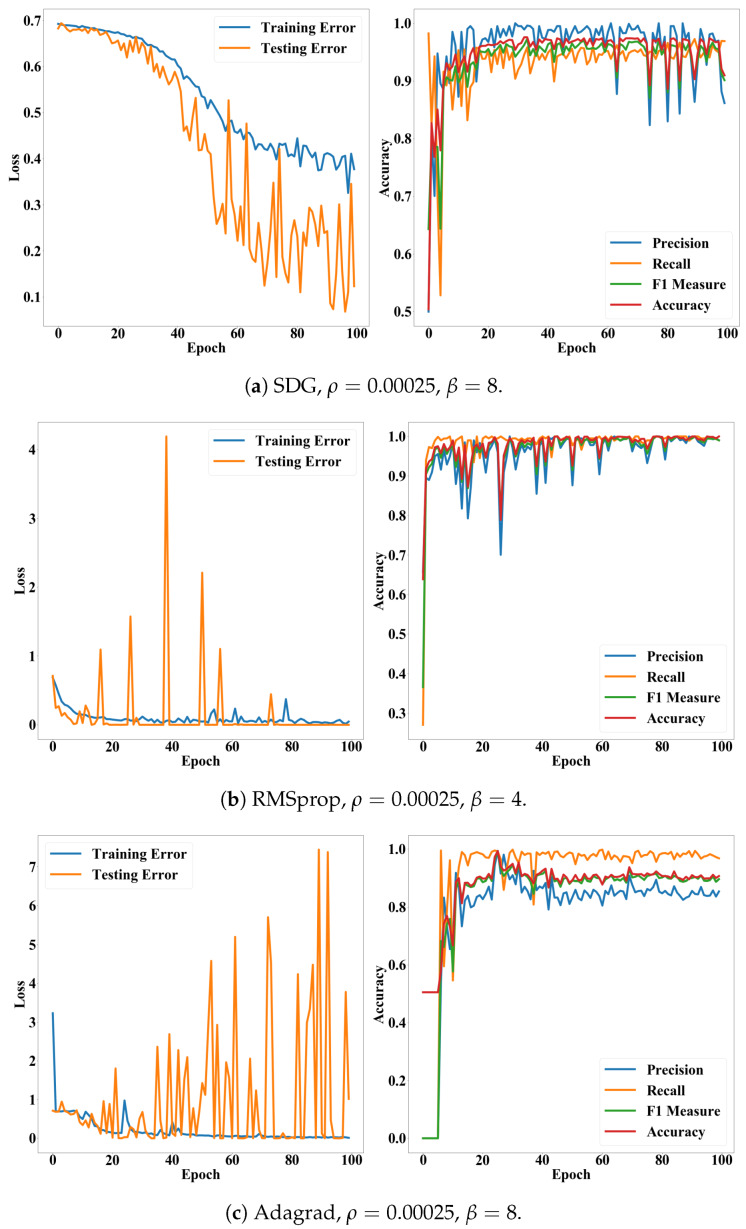
Loss and accuracy of the CNN model according to epochs. (**a**–**c**) present accuracy and loss of the SDG, RMSprop, and Adagrad on their optimal hyper-parameters, respectively

**Figure 9:**
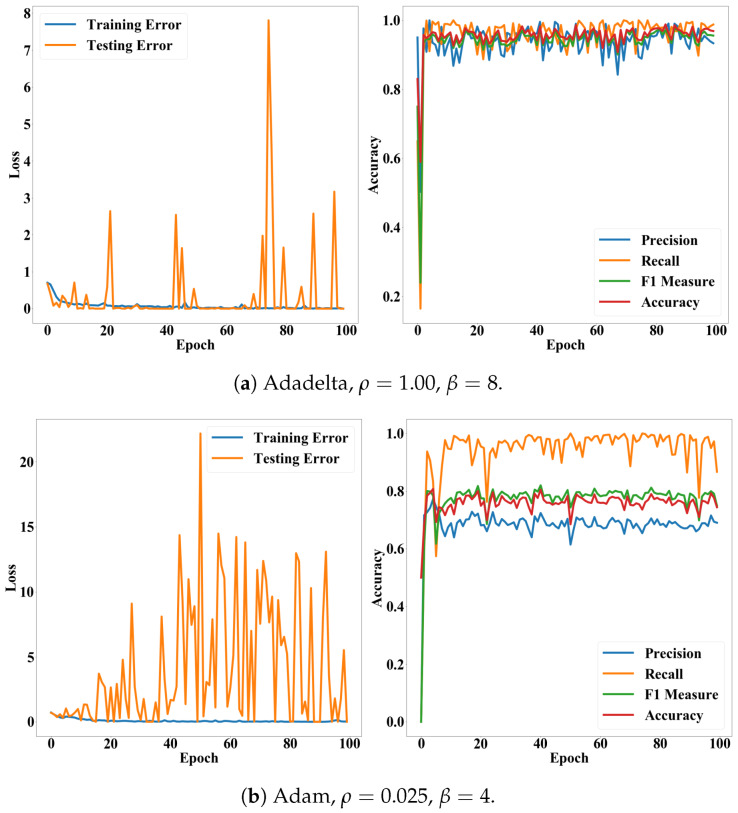
Loss and accuracy of the CNN model according to epochs. Subfigures (**a**,**b**) present accuracy and loss of the Adadelta and Adam on their optimal hyper-parameters, respectively.

The batch size indicates how frequently we update the weights of our CNN model. When the batch size is 2, we update the weights according to the loss of every two images in the training set. One epoch indicates one iteration of training. Thus, ϵ denotes how many iterations we will conduct. There are various methods for searching optimal weights θ [36]. We applied five methods: stochastic gradient descent (SGD) [36], RMSprop (http://www.cs.toronto.edu/~tijmen/csc321/slides/lecture_slides_lec6.pdf), Adagrad [37], Adadelta [38], and Adam [39]. Since Adadelta applies a decay factor to the learning rate according to epochs and recommends setting the initial learning rate as 1.00, we did not search the optimal learning rate for the Adadelta. We also conducted the hyper-parameter search for all the methods. Performances of the CNN model, according to epochs, exhibit stability of the proposed model. Figure 8 and Figure 9 show performance fluctuations of the optimizers on the same validation case. We compared the optimizers using their accuracy and loss on the optimal hyper-parameters and epoch. We employed the binary cross-entropy loss ∈[0,1] (Equation (7) in Section 4.6.2). Table 2 presents averages and standard deviations of the performance metrics over the validation cases.

We also examined whether the proposed method is applicable to the calcium fluorescence live-cell images, which are widely used for tracking the oscillation of cytosolic calcium concentration. Fluorescent intensity caused by intracellular calcium also plays a fundamental role in determining cancer invasiveness. It is worthwhile to note that Figure 4 and Figure 5 demonstrate that the deformability and fluorescence intensities of MDA-MB-231 are significantly higher than that of MCF-7, which is in agreement with existing literature [40,41]. We took a photomicrograph of 10 fluorescent cells consisting of five MDA-MB-231 and five MCF-7 cells. We assessed whether the proposed model, which is trained for non-fluorescent cells, can be used for fluorescent cells. This experiment can validate network generalization of the proposed model by showing that the model is capable of handling the diversity of cell photomicrographs. We used models trained by RMSprop and Adadelta optimizers, which have the highest accuracy. Table 3 presents the performance metrics for fluorescence cell images.

## 3. Discussion

In this study, we assumed that the conventional CNN model would be enough to classify cells according to their deformations. As shown in Table 2, the CNN model exhibited remarkably high accuracy. With the Adadelta, the most suitable optimizer, accuracy, precision, recall, and $F_{1}$ measure of the model were commonly greater than 0.96. The accuracy was also stable for both validation cases and epochs. Particularly, the Adadelta and RMSprop performed significantly lower variances than the other cases. Their recall was 0.99 ± 0.01. Their standard deviations for the accuracy and $F_{1}$ measure were 0.05. This result underpins the finding that we can diagnose diseases, which affect the deformations of cells, quickly and automatically, by integrating the ultrasonic devices and CNN model.

In addition, Figure 8 and Figure 9 show that the accuracy and $F_{1}$ measure of the proposed model were more stable than the precision and recall while converging according to epochs. In general, the precision and recall have tended to show a trade-off relationship, and our results in Figure 8 and Figure 9 also revealed the same, but the precision decreased more while the recall increased. Validation losses revealed these problems: (i) The proposed model exhibited significant fluctuations in their validation losses according to epochs, while the training loss converged. (ii) The Adadelta and RMSprop optimizers exhibited higher and less stable validation losses than the other optimizers. The high loss with high accuracy in the binary cross-entropy indicates our model generated correct answers, but with low confidence. In other words, the cell deformation was an effective feature for diagnosing invasiveness of cancers automatically, but borderline cases still exist. This issue will be resolved by employing additional features or data samples as shown in the fluorescence experiments (Table 3).

Although the difference between the SBAT on and off images was much more vivid in the fluorescence cases than in the original cases (Figure 4 and Figure 5), the accuracy of the proposed method was lower in the fluorescence images. This is because the proposed model was not trained for the fluorescence images. Yet, the RMSprop optimizer still exhibited high precision (0.97), $F_{1}$ measure (0.89), accuracy (0.90), which represents that the proposed model is applicable for both fluorescent and non-fluorescent cells. On the fluorescence images, their precision was commonly higher than their recall. It implies that these images showed not only the deformation caused by the SBAT but also brightness changes. As we expected, additional features were helpful for dealing with the borderline cases.

Additionally, the experimental results show that the proposed model was free from the overfitting issue. Although the number of samples is restricted, we exhibited that our CNN model had reliable and stable performances on overall samples by using the *k*-fold cross-validation. The experiment of fluorescence images also supports that the proposed model can handle the diversity of photomicrographs produced in this research domain. Moreover, by adopting the shallow CNN, we attempted to avoid the possibility of the overfitting and showed that the shallow model is enough to classify cancer cells according to their invasiveness. At this moment, we are not sure that the proposed model is generally applicable to other cancer cell lines or diseases. Nevertheless, the experimental results are enough to show the necessity and prominence of integrating the SBAT and machine learning techniques.

In summary, this study experimentally demonstrated the capability of the SBAT to deform the cell and to classify the breast cancer cell based on their invasiveness through CNN. It was shown that the proposed model exhibited reasonable accuracy for both non-fluorescent and fluorescent and cells. Typically, the images CNN trained in this study is quite common, i.e., the cell morphology and background. Therefore, the relatively lower recall than its precision was found and might be caused by not offering various features for training. To enhance the recall rate of CNN, the sample number and the types of cells can be increased.

## 4. Material and Method

### 4.1. Transducer Fabrication

Piezoelectric single crystals, lithium niobate (LiNbO${}_{3}$) are widely used to manufacture high-frequency ultrasonic transducers due to its high electromechanical coupling coefficient ($k_{t}∼49$%) and low dielectric permittivity ($\epsilon^{s}∼39$). We fabricated a 50 MHz press-focused transducer using the 36${}^{\circ }$-rotated Y-cut LiNbO${}_{3}$ (Boston Piezo-Optics, Bellingham, MA, USA) with the following steps [16,21]. A Krimholtz, Leedom, and Matthaei model software (PiezoCAD, Sonic Concepts, Bothell, WA, USA) offered both an optimal aperture size and thickness of an acoustic stack which includes piezoelectric, matching, and backing layers. LiNbO${}_{3}$ was bonded to the glass plate and was manually lapped down to 61 μm. After the lapping process, chrome (500 Å) and gold electrodes (1000 Å) (Cr/Au, Nano-Master, Austin, TX, USA) were sputtered on the matching side of the material. The first matching layer, a mixture of 2–3 μm silver particles (silver; Aldrich Chemical Co., St. Louis, MO, USA) and Insulcast 501 epoxy (Insulcast 501, American Safety Technologies, Roseland, NJ, USA), was bonded to the front side of the LiNbO${}_{3}$ layer and lapped down to 9 μm. Chrome and gold electrodes were sputtered on the backing side of the LiNbO${}_{3}$ layer. Conductive epoxy (E-solder 3022, Von Roll Isola, Schenectady, NY, USA) was attached to the backside of the material at a thickness of 1 mm, and the final acoustic stack is fabricated. After the acoustic stack was turned down to a diameter of 5 mm using a lathe, it was wired with a single-lead wire at the backing layer. The stack was concentrically placed in a brass housing. The gap between the acoustic stack and the housing was filled with an insulating epoxy (Epo-tek 301, Epoxy Technologies, Billerica, MA, USA) to prevent a short circuit. A heated bearing ball was placed on the surface of the matching layer and mechanically pressed to generate a concave structure. Another layer of chrome and gold electrodes with a thickness of 1500 Å was sputtered on top to make a ground signal. An SMA electrical connector was mounted, and the second matching layer, a parylene film (10.5 μm), was coated the outermost surface of the transducer using a PDS 2010 Labcoater (SCS, Indianapolis, IN, USA) for the second matching layer and protection purposes.

### 4.2. Transducer Performance

A JSR pulser/receiver (DPR500, Pittsford, NY, USA) was used for a pulse-echo test of the fabricated transducer. It generated electrical impulses at a 500 Hz repetition rate and a damping ratio of 50. RF echo signals of the transducer received from a flat quartz reflector were analyzed. Figure 2a,b shows a measured pulse-echo response and the frequency spectrum, respectively. The center frequency was 50 MHz, and the −6 dB fractional bandwidth was 80%. Quantitative spatial peak temporal average intensity (I${}_{\mathrm{SPTA}}$) in two-dimensional lateral and axial directions was derived after calibration with a needle hydrophone (Precision Acoustics, UK) as shown in Figure 2c. The driving conditions were as follows: frequency of 50 MHz, pulse repetition frequency (PRF) of 1 kHz, cycle number of 10, and input peak to peak voltage of 25 V. The −3 dB lateral beam width was measured to be 32 μm. Lateral resolution is determined by the center frequency and f-number of the transducer. F-number (the focal distance of 4 mm/aperture diameter of 5 mm) was calculated to be 0.8, and the theoretical lateral resolution is 24 μm.

### 4.3. Cell Preparation

Human breast cancer cell lines, MDA-MB-231 and MCF-7, were purchased from ATCC (Manassas, VA, USA) and maintained in modified complete medium (RPMI, 10% fetal bovine serum, 10 mM HEPES, 2 mM L-glutamine, 1 mM sodium-pyruvate, 0.05 mM 2-mercaptoethanol, and 11 mM D-glucose). They were cultured in 5% CO${}_{2}$ at 37 ${}^{\circ }$C. The SBAT traps and deforms a single-cell in a suspended cell, so a trypsin-ethylenediaminetetraacetic acid (trypsin-EDTA) solution obtained from Invitrogen (Grand Island, NY, USA) was used to detach cultured cells from the bottom of the Petri dish. After the addition of trypsin-EDTA into the culture dish, the cells were incubated at 37 ${}^{\circ }$C for 2 min. An equivalent volume of modified complete medium was added to neutralize the trypsin. Phosphate buffer solution (PBS) was purchased from Invitrogen (Grand Island, NY, USA) for washing cells before acoustic tweezer experiments. With the inverted microscope, we confirmed that the cell was slightly touching or floating on the Petri dish without morphological damage. During experiments, cells with blebs were excluded from the sample for measurements. The cell viability test also validated that there was no significant adverse effect on the cell’s condition during the experiment.

### 4.4. Live Intracellular Calcium Imaging

For the fluorescence cell image, both cell lines of MDA-MB-231 and MCF-7 were seeded on culture dishes and kept in the CO${}_{2}$ incubator for 48 h before experiments. HBSS with Ca${}^{2+}$ and Mg${}^{2+}$ was used as the working solution. Cells were washed with HBSS three times and incubated with 3 μm of Fluo-4 AM at room temperature for 30 min for Ca${}^{2+}$ imaging. After incubation, the cells were washed three times with HBSS and imaged with an epi-fluorescence inverted microscope during experiments.

### 4.5. SBAT for Cell Deformation

The demonstration of an acoustic tweezers system is described in Figure 1. A focused 50 MHz ultrasonic transducer and ultrasound electronics which includes a pulser–receiver, a function generator (Stanford Research Systems, Sunnyvale, CA, USA), and a 50 dB power amplifier (525LA, ENI, Rochester, NY, USA) were integrated with an inverted fluorescence microscope (Olympus IX-71, Center Valley, PA, USA) to monitor the SBAT. The movement of the transducer was controlled by a three-axis motorized stage (SGSP 20, Sigma KOKI Co., Midori, Tokyo, Japan). The focal point on the Petri dish was aligned using a pulser–receiver, and a 50 MHz sinusoidal burst signal, generated by a function generator and amplified by a power amplifier, was driven on the transducer to trap, manipulate, and deform a suspended single-cell. The duty cycle and PRF were set to 500 cycles and 1 kHz, respectively. The input peak-to-peak voltage was set to 0.00, 4.74, 9.48, 14.22, 18.96, or 23.70 $V_{pp}$ (corresponding acoustic pressures: 0.00, 0.23, 0.43, 0.63, 0.82, and 1.00 MPa, respectively). An inverted microscope and a CMOS camera (ORCA-Flash2.8, Hamamatsu, Japan) were used for the recording of the SBAT and cell deformation.

### 4.6. Cancer Cell Classification with Convolutional Neural Networks

The study aim was to validate whether invasive cancer cells can be detected automatically using a conventional CNN model. We applied the CNN model to 40 cells. Half of the cells had significant deformation and invasiveness (MDA-MB-231), and the half did not (MCF-7). For each cell, we took photos with the SBAT on and off. Then, the CNN model was trained to classify cancer cells into invasive and non-invasive groups. The deformation is a major feature of the classification. However, since CNN is one of the black box models, our model will learn various and uninterpretable features from cell images.

#### 4.6.1. Preprocessing

In this study, we use a conventional CNN model, which cannot deal with time-serial data. However, since we expect that the deformation will be the key feature, the model has to consider changes in cell size. We propose an image preprocessing method to solve this problem. Most of the image files consist of multiple color channels (e.g., red, green, and blue channels). The CNN model also accepts multi-channel images. On the other hand, our input images (photomicrographs) are gray-level images. Thus, we deliver cell images with the SBAT on and off indicating the different cell deformability through the red and green channels of the input image, respectively, as shown in Figure 3.

Detail procedures of the preprocessing are as follows.

1. Enhance contrast of cell images.
2. Put the SBAT on images as the red channel, the SBAT off images as the green channel, and the average of the SBAT on and off images as the blue channel.
3. Save the combined image.

Since some cell images include noise from reflected light, CNN model is taught to recognize the noise by using two methods. First, cell areas and boundaries are emphasized by enhancing the contrast of the images. The enhancement is conducted by normalizing pixel values of the images into [0,255]. This can be formulated as:

(2) $$\begin{array}{c} p_{x,y}^{*}=\left\lceil \frac{p_{x,y}-\min_{\forall i,j}p_{i,j}}{\max_{\forall i,j}p_{i,j}-\min_{\forall i,j}p_{i,j}}\times 255\right\rceil \end{array}$$

where $p_{x,y}$ is a pixel value, (x,y) indicates a pixel coordinate, $p_{x,y}^{*}$ denotes a pixel value after the contrast enhancement, and ⌈·⌉ denotes the rounding function.

Second, for the machines, it is difficult to identify which parts of images are cells or backgrounds. Changes between the SBAT ‘off’ and ‘on’ images mainly occur in the cells. Thus, we made a new channel by averaging corresponding pixel values from the two images. The average will dilute the changes and preserve only the backgrounds. This can be formulated as:

(3) $$\begin{array}{c} p_{x,y}^{N}=\left\lceil \frac{1}{2}\times \left(p_{x,y}^{B}+p_{x,y}^{A}\right)\right\rceil \end{array}$$

where $p_{x,y}^{N}$, $p_{x,y}^{B}$, and $p_{x,y}^{A}$ are pixels on (x,y) in the ‘background,’ ‘SBAT off,’ and ‘SBAT on’ channels, respectively. Therefore, on our input image (I=〈B,A,N〉), cells with the SBAT on and off are marked by red and green colors, respectively, as displayed in Figure 3. Figure 4 shows a comparison of preprocessing results of MDA-MB-231 cells with MCF-7 cells. For fluorescent cell images, we can use the same preprocessing methods. Figure 5 presents preprocessing results for the MDA-MB-231 and MCF-7 cells dyed by the fluorescent pigments. The deformation was more visible in the fluorescent cells than in the non-fluorescent ones.

Additionally, we took 40 pairs of images from the 40 cells. However, the number of samples is not enough to train the CNN model. Thus, image augmentation was conducted. We employed the augmentation tool supported by Keras (https://keras.io/preprocessing/image/#imagedatagenerator-class). The augmentation tool generates new images by rotating, scaling, and translating the original images, as displayed in Figure 6. This process also makes the CNN model robust to those transformations. When the proposed model is deployed, the quality of the input photomicrographs is not guaranteed. Operators of this model cannot always be well-trained experts. Therefore, robustness is significant for the practicality of the proposed model.

#### 4.6.2. CNN Model for Cancer Cell Classification

We applied the conventional CNN model to detect invasive cancer cells. We expected that the conventional model would be enough for this task, since deformability, our key feature, is vivid in the preprocessing results (Figure 4 and Figure 5). In this research domain, it is difficult to collect an enormous amount of cell images to train deep CNN models that consist of hundreds of convolutional layers. Although we use the data augmentation, the deep models include too many weights to avoid the overfitting issue. In Section 2 and Section 3, we exhibited that the shallow model has enough accuracy and is free from the overfitting by using the *k*-fold cross-validation and fluorescence cell images. This model consists of three two-dimensional convolutional layers, three max-pooling layers, and two FC layers. After the convolution parts, we flattened the outputs from matrices to a vector. Then, we put the vector as the input of the FC layers. Lastly, based on the output of the FC layers, we classified cells into two groups: MDA-MB-231 and MCF-7 cells. Figure 7 presents the structure of the CNN model in detail.

The convolutional layers consist of multiple convolutional filters. For example, our first convolutional layer consists of thirty-two 3×3 convolutional filters. The filters are square matrices, and their elements are weighting factors. Each convolutional filter calculates the weighted summation of pixel values in a part of the input image. The weighted summation reflects visual features in the part. Conventionally, the filters were designed to detect particular visual features using gradients of pixel values. For example, to detect horizontal edges, we can contrast pixels on the upper sides with on lower sides. This can be formulated as follows:

(4) $$\begin{array}{c} \left[\begin{array}{ccc} 0 & 0 & 0\\ 0 & 0 & 0\\ 10 & 10 & 10 \end{array}\right]*\left[\begin{array}{ccc} 1 & 1 & 1\\ 0 & 0 & 0\\ -1 & -1 & -1 \end{array}\right]=-30,\;\;\left[\begin{array}{ccc} 10 & 10 & 10\\ 10 & 10 & 10\\ 10 & 10 & 10 \end{array}\right]*\left[\begin{array}{ccc} 1 & 1 & 1\\ 0 & 0 & 0\\ -1 & -1 & -1 \end{array}\right]=0 \end{array}$$

where ∗ denotes the weighted summation, the first matrices are parts of the input image, and the second matrix is a filter for detecting horizontal edges. The filter searches the input image by calculating the weighted summation on every *n* pixels. The step size *n* is called as stride, and the filter moves *n* pixels vertically or horizontally from top-left corner to bottom-right corner of the input image. However, there are limitations to design all the filters heuristically. Especially, shapes and deformation of the cells are not much typical. In other words, we expect that the deformation is a distinctive feature of cancer cells. Nevertheless, it is a challenging task to design convolutional filters for detecting the deformation over the noisy and atypical photomicrographs. Thus, we employed a CNN that can train convolutional filters for a specific purpose with a black-box approach.

Simply speaking, we can express the layers in the CNN model, which consist of a number of convolutional filters, as linear functions. Each layer calculates weighted summations of input variables and applies activation functions on the summations. Outputs of lower layers are inputs of the upper layers. Since the linear functions are too simple to solve complicated problems, the activation functions transform output spaces of neural networks into non-linear spaces. All the layers of our model, excluding the output layer, use the rectified linear unit (ReLu) function as their activation, which is most widely used. The output layer uses the sigmoid function for the binary classification. Thus, our model f(·) can be formulated as:

(5) $$\begin{array}{ccc} f(X;\theta ) & = & f^{\left(5\right)}\left(f^{\left(4\right)}\left(\cdots f^{\left(1\right)}\left(X;\theta_{1}\right)\cdots \right)\right), \end{array}$$

(6) $$\begin{array}{ccc} f^{\left(n\right)}\left(X;\theta \right) & = & h^{\left(n\right)}\left(\theta_{n}^{⊺}X+b\right) \end{array}$$

where $f^{\left(n\right)}\left(\cdot \right)$ denotes the *n*-th layer, $h^{\left(n\right)}\left(\cdot \right)$ refers to the activation function of the *n*-th layer, $\theta_{n}$ indicates weights on the *n*-th layer.

The training is conducted by the back propagation. Errors on the results of the CNN model are propagated to all the convolutional filters to update their weightings. Since our task is binary classification, we use the binary cross-entropy function to measure the loss (error) of our model. The output of the model is a real number in [0,1]. When the output is greater than 0.5, we determine that a cell in the input image is in the MDA-MB-231 group; otherwise, in the MCF-7. Therefore, we train the model to print 1 for the MDA-MB-231 cells and 0 for the others. The loss function can be formulated as:

(7) $$\begin{array}{c} L\left(\theta \right)=\frac{1}{N}\times \sum_{\forall X}Y\times \log f\left(X;\theta \right)+\left(1-Y\right)\times \log f\left(X;\theta \right) \end{array}$$

where *Y* is the ground truth for the input image *X*, which is 1 for the MDA-MB-231 cells and 0 for the others. When the model makes correct answers, L(θ) will be 0; otherwise, positive real numbers.

We train the model to find the optimal weights that minimize L(θ). This optimization is conducted by using gradients of L(θ) to the weights θ. To update the weights, most of the optimization methods move the weights according to the directions and sizes of the gradients, with an assumption that L(θ) is a convex function. This can be formulated as:

(8) $$\begin{array}{c} \theta^{*}:=\theta -\rho \times \nabla L\left(\theta \right) \end{array}$$

where $\theta^{*}$ is the updated weights, and ρ denotes the learning rate, which means how rapidly the weights are updated. For larger gradients and learning rates, θ moves more quickly. The learning rate, ρ must be tuned not to be stuck in local optima and not to pass over convex areas. In Section 2, we searched for the optimization method and hyper-parameters appropriate for our model.

### 4.7. Cell Viability Test

**Figure 10:**
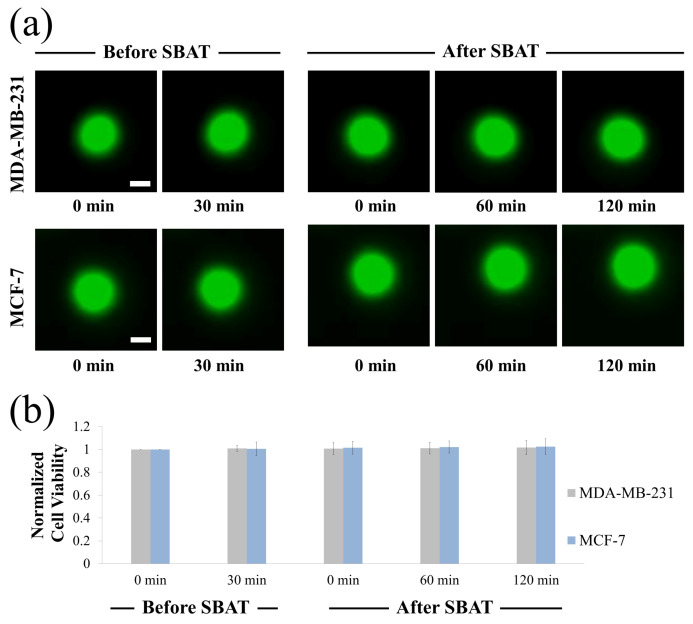
Cell viability assay upon single-beam acoustic tweezers (SBAT). (**a**) Fluorescence images of MDA-MB-231 and MCF-7 before SBAT (negative control) and after SBAT (experimental group). (**b**) Normalized fluorescence intensity of cells before and after SBAT. Error bars indicate standard deviations. The values at each cell line are the average of 20 samples. Scale bars indicate 10 μm. There were no significant effects on the cell condition after SBAT.

Cytotoxicity of SBATs on MDA-MB-231 and MCF-7 cells was evaluated using Calcein-AM (Thermo Fisher Scientific, Indianapolis, IN, USA). Calcein-AM is a dye that enters live cells converting to green fluorescent. Calcein-AM was prepared as a stock solution of 1 mM in dimethylsulfoxide at room temperature. A final concentration of 10 μm of Calcein-AM was added into the cell culture dish. Fluorescence imaging of the cells was observed using a microscope (an excitation of 488 nm and an emission of 532 nm). Figure 10 shows the results of the cell viability experiments: before SBAT (negative control) and after SBAT (experiment). The cells exposed to SBAT with 1.0 MPa for 1 min. If ultrasound affects the cell membrane integrity, the decrease of fluorescence intensity is observed. The normalized mean viabilities (0, 60, 120 min after trapping) for the MDA-MB-231 and MCF-7 cells were 1.012 ± 0.039 and 1.020 ± 0.038, respectively. The values at each cell line are the average of 20 samples. The *p*-values of all three cell groups were greater than 0.05 (p= 0.822 and 0.624 for MDA-MB-231 and MCF-7, respectively). No significant sign of cytotoxicity was found in both the non-trapping and trapping groups.

## 5. Conclusions

We demonstrated that SBAT with CNN based image analysis could serve as a platform for cancer cell evaluation. The high-frequency SBAT is a non-contact and non-labeling technique for the trapping and mechanically deforming of micron-sized objects such as particles or cells. The deformation of MDA-MB-231 and MCF-7 cells in vitro using the SBAT was successfully demonstrated in the paper. Previous methods to evaluate the invasive potential of cancer cells, such as manual analysis, are time-consuming and subjective. CNN clearly provides the classification between two cell lines, highly and weakly invasive cancer cells, based on pretraining and optimization. As a result, high precision and recall rates (>0.96) of the model have been achieved. For further development of the integrated SBATs and CNN, this system can be used for automatically estimating the elastic modulus of cancer cells by applying image processing techniques on cell photomicrographs. After image segmentation into cell and background areas, it is possible to measure ratios of changes in cell areas automatically. Then, CNN processes the correlation between cell deformability and acoustic pressure. Other than the mechanical deformation of a cancer cell, the calcium ion dynamics of a cell evoked by the SBAT is another important indicator for determining the cell invasiveness and its mechanotransduction pathway. This system can perform the automatic analysis of ultrasound-induced calcium elevation for a better understanding of various cellular functions.

## Figures and Tables

**Table 1:** Ranges and step sizes for the hyper-parameter search.

Parameter	Range	Step Size
Learning rate (ρ)	[0.25,0.00025]	×10
Batch size (β)	[1,32]	×2
Epochs (ϵ)	[20,300]	+20

**Table 2:** The best performance of the proposed CNN model on each optimizer. Values in round brackets are standard deviations of the performance indicators for all validation cases. * and ** mark the first and second best performance, respectively.

Optimizer	Validating Loss	Accuracy	Precision	Recall	$F_{1}$ Measure
SDG	0.25 ** (0.20 *)	0.91 (0.06)	0.92 (0.07 *)	0.91 (0.08)	0.90 (0.07)
RMSprop	0.89 (1.65)	0.96 ** (0.05 **)	0.93 ** (0.08)	0.99 * (0.01 *)	0.95 ** (0.05 **)
Adagrad	0.21 * (0.25 **)	0.85 (0.19)	0.83 (0.19)	0.98 (0.02)	0.88 (0.13)
Adadelta	1.51 (2.94)	0.97 * (0.05 *)	0.96 * (0.08 **)	0.99 ** (0.01 **)	0.97 * (0.05 *)
Adam	0.57 (0.71)	0.88 (0.09)	0.86 (0.13)	0.93 (0.07)	0.88 (0.09)

**Table 3:** Performance of the proposed CNN model for the fluorescence cell images.

Optimizer	Validating Loss	Accuracy	Precision	Recall	$F_{1}$ Measure
RMSprop	0.00	0.90	0.97	0.84	0.89
Adadelta	8.49	0.85	0.87	0.82	0.82

## References

[1] Zhang N., Wu J., Yang M., Yu J., Li R. (2019). A Composite Model Integrating Imaging, Histological, and Genetic Features to Predict Tumor Mutation Burden in Non-Small Cell Lung Cancer Patients. Int. J. Radiat. Oncol. Biol. Phys..

[2] Rossi G., Berbellini A., Ancidei S., Montini G.C., Capoccetti F., Cidda C., Fattori S., Cardarelli M., Patrizi I., Brianzoni E. (2015). Calculation of Lesion Volume in Non-Small Cell Lung Cancer (NSCLC) by PET/CT Imaging: Histological Comparation and Threshold Study. Preliminary Report. Eur. J. Nucl. Med. Mol. Imaging.

[3] Paschali A., Papandrianos N., Koletsis E., Stamou E., Spyridonidis T., Savvopoulos C., Barla P., Dougenis D., Vassilakos P.J., Apostolopoulos D.J. (2009). The Value Of 99mTc-Depreotide SPECT/CT Imaging For Lymph Node Staging In Non-Small-Cell Lung Cancer. A Study With Histological Confirmation. Eur. J. Nucl. Med. Mol. Imaging.

[4] Gallastegui A., Cheung J., Southard T., Hume K.R. (2018). Volumetric and linear measurements of lung tumor burden from non-gated micro-CT imaging correlate with histological analysis in a genetically engineered mouse model of non-small cell lung cancer. Lab. Anim..

[5] Kurc T., Sharma A., Oh T., Farris A., Saltz J., Wang F., Cooper L., Kong J., Pan T., Chen W. (2011). A data model and database for high-resolution pathology analytical image informatics. J. Pathol. Inform..

[6] Nelissen B.G.L., van Herwaarden J.A., Moll F.L., van Diest P.J., Pasterkamp G. (2014). SlideToolkit: An Assistive Toolset for the Histological Quantification of Whole Slide Images. PLoS ONE.

[7] Zerbe N., Hufnagl P., Schlüns K. (2011). Distributed computing in image analysis using open source frameworks and application to image sharpness assessment of histological whole slide images. Diagn. Pathol..

[8] Li Q., Lee G., Ong C., Lim C. (2008). AFM indentation study of breast cancer cells. Biochem. Biophys. Res. Commun..

[9] Cross S.E., Jin Y.S., Tondre J., Wong R., Rao J., Gimzewski J.K. (2008). AFM-based analysis of human metastatic cancer cells. Nanotechnology.

[10] Rico F., Roca-Cusachs P., Gavara N., Farré R., Rotger M., Navajas D. (2005). Probing mechanical properties of living cells by atomic force microscopy with blunted pyramidal cantilever tips. Phys. Rev. E.

[11] Coceano G., Yousafzai M.S., Ma W., Ndoye F., Venturelli L., Hussain I., Bonin S., Niemela J., Scoles G., Cojoc D. (2015). Investigation into local cell mechanics by atomic force microscopy mapping and optical tweezer vertical indentation. Nanotechnology.

[12] Grier D.G. (2003). A revolution in optical manipulation. Nature.

[13] Walter N., Selhuber C., Kessler H., Spatz J.P. (2006). Cellular Unbinding Forces of Initial Adhesion Processes on Nanopatterned Surfaces Probed with Magnetic Tweezers. Nano Lett..

[14] Yoon J.K., Lee T.I., Bhang S.H., Shin J.Y., Myoung J.M., Kim B.S. (2017). Stretchable Piezoelectric Substrate Providing Pulsatile Mechanoelectric Cues for Cardiomyogenic Differentiation of Mesenchymal Stem Cells. ACS Appl. Mater. Interfaces.

[15] Melzer J.E., McLeod E. (2018). Fundamental Limits of Optical Tweezer Nanoparticle Manipulation Speeds. ACS Nano.

[16] Lim H.G., Li Y., Lin M.Y., Yoon C., Lee C., Jung H., Chow R.H., Shung K.K. (2016). Calibration of Trapping Force on Cell-Size Objects From Ultrahigh-Frequency Single-Beam Acoustic Tweezer. IEEE Trans. Ultrason. Ferroelectr. Freq. Control.

[17] Lim H.G., Kim H.H., Yoon C. (2018). Evaluation method for acoustic trapping performance by tracking motion of trapped microparticle. Jpn. J. Appl. Phys..

[18] Lim H.G., Kim H.H., Yoon C., Shung K.K. (2020). A One-Sided Acoustic Trap for Cell Immobilization Using 30-MHz Array Transducer. IEEE Trans. Ultrason. Ferroelectr. Freq. Control.

[19] Lam K.H., Li Y., Li Y., Lim H.G., Zhou Q., Shung K.K. (2016). Multifunctional single beam acoustic tweezer for non-invasive cell/organism manipulation and tissue imaging. Sci. Rep..

[20] Hwang J.Y., Yoon C.W., Lim H.G., Park J.M., Yoon S., Lee J., Shung K.K. (2015). Acoustic tweezers for studying intracellular calcium signaling in SKBR-3 human breast cancer cells. Ultrasonics.

[21] Lim H.G., Shung K.K. (2017). Quantification of Inter-Erythrocyte Forces with Ultra-High Frequency (410 MHz) Single Beam Acoustic Tweezer. Ann. Biomed. Eng..

[22] Liu H.C., Gang E.J., Kim H.N., Lim H.G., Jung H., Chen R., Abdel-Azim H., Shung K.K., Kim Y.M. (2018). Characterizing Deformability of Drug Resistant Patient-Derived Acute Lymphoblastic Leukemia (ALL) Cells Using Acoustic Tweezers. Sci. Rep..

[23] Hwang J.Y., Kim J., Park J.M., Lee C., Jung H., Lee J., Shung K.K. (2016). Cell Deformation by Single-beam Acoustic Trapping: A Promising Tool for Measurements of Cell Mechanics. Sci. Rep..

[24] Lee J., Lee C., Shung K.K. (2010). Calibration of sound forces in acoustic traps. IEEE Trans. Ultrason. Ferroelectr. Freq. Control.

[25] Lee J., Jeong J.S., Shung K.K. (2013). Microfluidic acoustic trapping force and stiffness measurement using viscous drag effect. Ultrasonics.

[26] Li Y., Lee C., Lam K.H., Shung K.K. (2013). A simple method for evaluating the trapping performance of acoustic tweezers. Appl. Phys. Lett..

[27] Hwang J.Y., Lim H.G., Yoon C.W., Lam K.H., Yoon S., Lee C., Chiu C.T., Kang B.J., Kim H.H., Shung K.K. (2014). Non-contact High-Frequency Ultrasound Microbeam Stimulation for Studying Mechanotransduction in Human Umbilical Vein Endothelial Cells. Ultrasound Med. Biol..

[28] Kim M.G., Park J., Lim H.G., Yoon S., Lee C., Chang J.H., Shung K.K. (2017). Label-free analysis of the characteristics of a single cell trapped by acoustic tweezers. Sci. Rep..

[29] Hwang J.Y., Lee C., Lam K.H., Kim H.H., Lee J., Shung K.K. (2014). Cell membrane deformation induced by a fibronectin-coated polystyrene microbead in a 200-MHz acoustic trap. IEEE Trans. Ultrason. Ferroelectr. Freq. Control.

[30] Calzado-Martín A., Encinar M., Tamayo J., Calleja M., Paulo A.S. (2016). Effect of Actin Organization on the Stiffness of Living Breast Cancer Cells Revealed by Peak-Force Modulation Atomic Force Microscopy. ACS Nano.

[31] Nikkhah M., Strobl J.S., Schmelz E.M., Agah M. (2011). Evaluation of the influence of growth medium composition on cell elasticity. J. Biomech..

[32] Wang B., Guo P., Auguste D.T. (2015). Mapping the CXCR4 receptor on breast cancer cells. Biomaterials.

[33] Cross S.E., Jin Y.S., Rao J., Gimzewski J.K. (2007). Nanomechanical analysis of cells from cancer patients. Nat. Nanotechnol..

[34] Darling E.M., Topel M., Zauscher S., Vail T.P., Guilak F. (2008). Viscoelastic properties of human mesenchymally-derived stem cells and primary osteoblasts, chondrocytes, and adipocytes. J. Biomech..

[35] Faria E.C., Ma N., Gazi E., Gardner P., Brown M., Clarke N., Snook R.D. (2008). Measurement of elastic properties of prostate cancer cells using AFM. Anal..

[36] Ruder S. (2016). An Overview of Gradient Descent Optimization Algorithms. arXiv.

[37] Duchi J., Hazan E., Singer Y. (2011). Adaptive Subgradient Methods for Online Learning and Stochastic Optimization. J. Mach. Learn. Res..

[38] Zeiler M.D. (2012). ADADELTA: An Adaptive Learning Rate Method. arXiv.

[39] Kingma D.P., Ba J. (2014). Adam: A Method for Stochastic Optimization. arXiv.

[40] Youn S., Choi J.W., Lee J.S., Kim J., Yang I.H., Chang J.H., Kim H.C., Hwang J.Y. (2019). Acoustic Trapping Technique for Studying Calcium Response of a Suspended Breast Cancer Cell: Determination of Its Invasion Potentials. IEEE Trans. Ultrason. Ferroelectr. Freq. Control.

[41] Hwang J.Y., Lee N.S., Lee C., Lam K.H., Kim H.H., Woo J., Lin M.Y., Kisler K., Choi H., Zhou Q. (2013). Investigating contactless high frequency ultrasound microbeam stimulation for determination of invasion potential of breast cancer cells. Biotechnol. Bioeng..

